# Species complex diversification by host plant use in an herbivorous insect: The source of Puerto Rican cactus mealybug pest and implications for biological control

**DOI:** 10.1002/ece3.6702

**Published:** 2020-08-20

**Authors:** Daniel Poveda‐Martínez, María Belén Aguirre, Guillermo Logarzo, Stephen D. Hight, Serguei Triapitsyn, Hilda Diaz‐Sotero, Marcelo Diniz Vitorino, Esteban Hasson

**Affiliations:** ^1^ Fundación para el Estudio de Especies Invasivas (FuEDEI) Hurlingham Argentina; ^2^ Instituto de Ecología Genética y Evolución de Buenos Aires (IEGEBA) Departamento de Ecología Genética y Evolución Universidad de Buenos Aires Buenos Aires Argentina; ^3^ Consejo Nacional de Investigaciones Científicas y Técnicas (CONICET) Ciudad Autónoma de Buenos Aires Argentina; ^4^ Grupo de investigación en Evolución, Ecología y Conservación (EECO) Universidad del Quindío Armenia Colombia; ^5^ U.S. Department of Agriculture‐ARS Tallahassee FL USA; ^6^ Department of Entomology University of California Riverside CA USA; ^7^ Caribbean Advisor to the APHIS Administrator USDA San Juan Puerto Rico; ^8^ Departamento de Engenharia Florestal Programa de Pós‐graduação em Engenharia Florestal ‐ PPGEF Lab. de Monitoramento e Proteção Florestal ‐ LAMPF Universidade Regional de Blumenau – FURB Blumenau Brazil

**Keywords:** cryptic species, host plants, *Hypogeococcus pungens*, insect pests, invasion, SNPs

## Abstract

Cryptic taxa have often been observed in the form of host‐associated species that diverged as the result of adaptation to alternate host plants. Untangling cryptic diversity in species complexes that encompass invasive species is a mandatory task for pest management. Moreover, investigating the evolutionary history of a species complex may help to understand the drivers of their diversification. The mealybug *Hypogeococcus pungens* was believed to be a polyphagous species from South America and has been reported as a pest devastating native cacti in Puerto Rico, also threatening cactus diversity in the Caribbean and North America. There is neither certainty about the identity of the pest nor the source population from South America. Recent studies pointed to substantial genetic differentiation among local populations, suggesting that *H. pungens* is a species complex. In this study, we used a combination of genome‐wide SNPs and mtDNA variation to investigate species diversity within *H. pungens* sensu lato to establish host plant ranges of each one of the putative members of the complex, to evaluate whether the pattern of host plant association drove diversification in the species complex, and to determine the source population of the Puerto Rican cactus pest. Our results suggested that *H. pungens* comprises at least five different species, each one strongly associated with specific host plants. We also established that the Puerto Rican cactus pest derives from southeastern Brazilian mealybugs. This is an important achievement because it will help to design reliable strategies for biological control using natural enemies of the pest from its native range.

## Graphical abstract

*Hypogeococcus pungens* is a species complex native to South America. Some of its members are cactophilic, and one of them has been reported as a pest threatening cactus diversity in the Caribbean and North America. Using a combination of genome‐wide SNPs and mtDNA, we found that the complex comprises at least five different species, each strongly associated with specific host plants. Our study also shows that the Puerto Rican cactus pest derives from southeastern Brazilian mealybugs. These results will help to design reliable strategies for biological control using natural enemies of the pest from its native range.

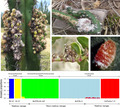

## 1 INTRODUCTION

Herbivorous insects are often involved in close interactions with their hosts since they are a food source and provide mating and oviposition sites (Schoonhoven, Van Loon, van Loon, & Dicke, 2005). Such intimacy often entails the evolution of adaptations that allow insects to cope with specific features of their host plants. Hence, host plant shifts may affect the evolution of features associated with feeding location, oviposition, and development on the host (Orsucci et al., 2018; Schoonhoven et al., 2005). Adaptation to new hosts may cause, either as a direct consequence or as a by‐product, the evolution of sexual isolation, highlighting the role of host plant shifts in speciation (Funk, Nosil, & Etges, 2006; Nosil, 2012).

Phytophagous insects with presumptively wide host ranges, that is, generalists, pose a concern from an applied view when dealing with insect pests. Many authors have suggested that polyphagous species are formed by locally adapted specialized populations or cryptic specialist species (e.g., Forbes et al., 2017; Loxdale & Harvey, 2016). A species complex made up of cryptic specialists pose an additional complication in defining host range. The distinction between true polyphagous and cryptic specialist species is crucial for the design of biological control programs (see below). In addition, cryptic species complexes offer the opportunity to investigate the role of host plants in the diversification of herbivorous insects since such complexes often include species of recent origin (Bakovic et al., 2019; Malka et al., 2018; Winter, Friedman, Astrin, Gottsberger, & Letsch, 2017).

Distinguishing between intra‐ and interspecific genetic variation is particularly relevant in cases of suspected cryptic species, especially when members of a guild of cryptic species are involved in invasions of new areas. Invasive insects may cause global problems upon spread to new territories, not only to agriculture, but also to biological diversity since invasive species are among the main causes of biodiversity loss (Newbold et al., 2015). The proper identification of invasive species is a necessary task to design successful biological control strategies to prevent or reduce harmful effects of invaders.

With the advent of molecular data, an increasing number of studies showed that some putative polyphagous insects are, actually, species complexes embracing several deeply diverged species, each one specialized to different host plant use (Egan, Nosil, & Funk, 2008; Nosil et al., 2012; Powell, Forbes, Hood, & Feder, 2014; but see Vidal, Quinn, Stireman, Tinghitella, & Murphy, 2019). So far, most population surveys have been based on the evaluation of a single genetic marker, the so‐called DNA‐barcoding gene encoding cytochrome oxidase subunit I (mtDNA) (Dinsdale, Cook, Riginos, Buckley, & De Barro, 2010; Stouthamer et al., 2017). However, it is well known that in many cases, this marker provides information limited to the mitochondrial lineage, potentially resulting in misidentification of species boundaries due either to incomplete lineage sorting or introgressive hybridization (Després, 2019; Eyer, Seltzer, Reiner‐Brodetzki, & Hefetz, 2017). Recently, population genetic studies benefited from availability of new methodologies based on high‐throughput sequencing of genomic libraries containing a reduced‐representation of nuclear genomes, known as genotype by sequencing, such as RADseq (Andrews, Good, Miller, Luikart, & Hohenlohe, 2016; Baird et al., 2008). These methodologies provide large multilocus datasets that can be used to evaluate cryptic diversity in species complexes (e.g., in Elfekih et al., 2018) and to trace the origin of invading pests (Anderson, Tay, McGaughran, Gordon, & Walsh, 2016; Ryan et al., 2019).

The cactus mealybug pest invading Puerto Rico and the adjacent smaller islands represents a threat for cactus diversity in the Caribbean, Central and North America. The pest was initially reported as *Hypogeococcus pungens* Granara de Willink (Hemiptera: Pseudococcidae), under the common name Harrisia cactus mealybug (HCM), the successful biological control agent released against an invasive cactus in Australia and South Africa (McFadyen & Tomley, 1978, 1981; Paterson et al., 2011). Currently, *H. pungens* sensu lato is considered a species complex, and in its native range (South America), it was reported to feed on species of Amaranthaceae, Portulacaceae, and Cactaceae (Ben‐Dov, 1994; Claps & Haro, 2001; Zimmermann, Pérez, Cuen, Mandujano, & Golubov, 2010). Recent studies based on molecular data and assessing reproductive compatibility (Poveda‐Martínez et al., 2019) and performance on different hosts plants (Aguirre et al., 2016) suggest that populations collected in Argentina, initially identified as *H. pungens* sensu lato, were, actually, a species complex comprising at least two species: one mealybug species feeding on Amaranthaceae (*H. pungens* sensu stricto) and the other an undescribed species feeding on cactus. Interestingly, the Puerto Rican cactus pest appears closer to *H. pungens* sensu stricto in phylogenetic trees suggesting that the pest shares a recent ancestor with the latter rather than with the sympatric, cactus feeding new species from Argentina. However, none of the mitochondrial haplotypes found in Argentina matched the single haplotype detected in the Puerto Rican mealybugs feeding on Cactaceae, suggesting that the source population of the pest was not Argentina (Poveda‐Martínez et al., 2019).

In the present study, we extended the sampling effort to a large geographic area comprising both the native (Argentina, Paraguay, and Brazil) and non‐native (Puerto Rico and southern United States) ranges by collecting mealybugs on host plants recognized as part of the diet of *H. pungens* sensu lato. We used a combination of genome‐wide SNPs and mtDNA variation to investigate: (a) genomic diversity within *H. pungens* species complex, (b) host plant ranges of each one of the putative members of the complex; (c) whether host plant shifts drove the diversification in the species complex, and (d) the source population of the Puerto Rican cactus pest. Based on these results, we will be able to search for and select biological control strategies using natural enemies, either those that co‐evolved with the pest (classical biological control) or those that did not co‐evolve but attack closely related species to the pest (new association biological control).

## 2 METHODS

### 2.1 Sample collections and DNA extraction

**FIGURE 1:**
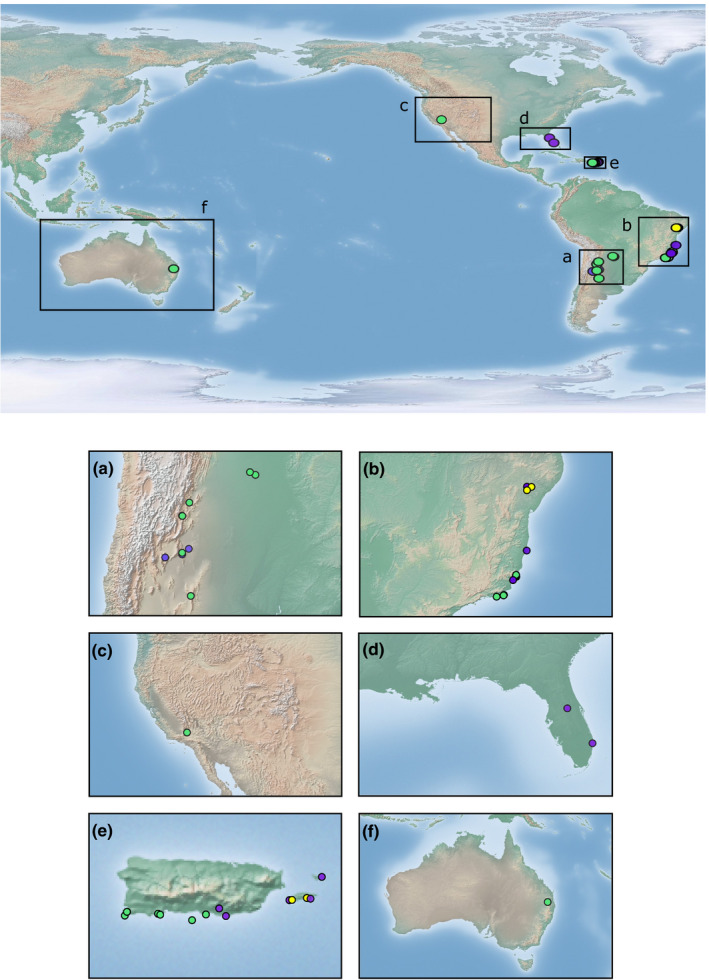
Locations of mealybug collection sites in both the native and non‐native range of *Hypogeococcus pungens* species complex. Green circles represent Cactaceae host plants where mealybugs were collected; purple circles represent Amaranthaceae host plants, while yellow circles represent Portulacaceae host plants. Panel (a) and (b) correspond with the native range distribution, while panel (c–f) correspond with the non‐native range distribution of the species complex

Samples of the mealybugs were collected in the native range of *H. pungens* sensu lato in three South American countries (*n* = 80) and in the recently invaded areas of Puerto Rico and the continental United States (*n* = 93). Mealybugs were collected on the host plants reported as part of the diet of *H. pungens* sensu* lato* (Cactaceae, *n* = 89; Amaranthaceae, *n* = 69; and Portulacaceae, *n* = 25). In the native range, mealybugs were collected in northern and northwestern Argentina (*n* = 21 on Cactaceae and *n* = 18 on Amaranthaceae), along the Atlantic coast of Brazil (*n* = 23 on Cactaceae; *n* = 11 on Amaranthaceae and *n* = 4 on Portulacaceae), and in western Paraguay (*n* = 3 on Cactaceae). We also included samples collected in the non‐native range in Puerto Rico (*n* = 42 on Cactaceae; *n* = 24 on Amaranthaceae, and *n* = 11 on Portulacaceae), southeastern United States (*n* = 11 on Amaranthaceae), and in southwestern United States (*n* = 5 on Cactaceae). Additionally, we included samples from southeastern Australia (*n* = 5 on Cactaceae), where *H. pungens* sensu lato from Argentina was introduced as a biological control agent against cactus weeds in the 1970s (McFadyen, 2012). All individuals were preserved in 100% ethanol and stored in a freezer until DNA extraction. Information concerning sampling localities, geographic coordinates, and host plants species is presented in Figure 1 and Table 1.

**TABLE 1:** List of mealybug populations sampled from native and non‐native ranges, their locations, geographic coordinates, sampling dates, and host plants on which specimens of the *Hypogeococcus pungens* species complex were collected

Population code	Country	Location	Host plant family	Host plant species	Latitude	Longitude	Collection date	No. indv
Native range distribution								
ARA	Argentina	Catamarca	Amaranthaceae	*Alternanthera pungens*	−28.57864	−65.53317	2011	5
ARA	Argentina	La Rioja	Amaranthaceae	*Alternanthera pungens*	−28.81261	−66.93668	2015	2
ARA	Argentina	La Rioja	Amaranthaceae	*Gomphrena* sp.	−28.80679	−66.93864	2015	5
ARA	Argentina	S. del Estero	Amaranthaceae	*Alternanthera pungens*	−28.14650	−65.12161	2011	3
ARA	Argentina	Salta	Amaranthaceae	*Alternanthera pungens*	−24.60278	−65.08123	2014	3
ARA	Argentina	Catamarca	Amaranthaceae	*Alternanthera paronychioides*	−28.7439	−65.54883	2016	1
ARC	Argentina	Salta	Cactaceae	*Cleistocactus baumannii*	−25.62661	−65.61704	2014	5
ARC	Argentina	Salta	Cactaceae	*Harrisia pomanensis*	−24.60278	−65.08123	2014	6
ARC	Argentina	Catamarca	Cactaceae	*Cleistocactus baumannii*	−28.44953	−65.63317	2018	5
ARC	Argentina	Cordoba	Cactaceae	*Cleistocactus smaragdiflorus*	−31.76076	−64.97155	2017	5
ARC	Paraguay	Boquerón	Cactaceae	*Cleistocactus baumannii*	−22.47643	−60.00823	2016	2
ARC	Paraguay	Boquerón	Cactaceae	*Cleistocactus baumannii*	−22.26690	−60.42901	2016	1
BRC	Brazil	Búzios	Cactaceae	unidentified	−22.74667	−41.87984	2015	3
BRC	Brazil	Búzios	Cactaceae	unidentified	−22.79990	−41.93037	2016	4
BRC	Brazil	Rio de Janeiro	Cactaceae	*Pilosocereus* sp.	−22.95672	−42.75225	2016	7
BRC	Brazil	Espírito Santo	Cactaceae	*Pilosocereus arrabidae*	−20.61087	−40.42837	2018	2
BRC	Brazil	Espírito Santo	Cactaceae	unidentified	−20.56176	−40.48261	2018	2
BRC	Brazil	Espírito Santo	Cactaceae	*Coleocephalocereus fluminensis*	−20.37727	−40.44545	2018	5
BRA	Brazil	Espírito Santo	Amaranthaceae	unidentified	−20.99056	−40.81029	2016	5
BRA	Brazil	Bahía	Amaranthaceae	*Alternanthera pungens*	−9.96483	−39.16336	2018	3
BRA	Brazil	Bahia	Amaranthaceae	unidentified	−9.99094	−38.63050	2018	1
BRA	Brazil	Bahia	Amaranthaceae	unidentified	−17.51244	−39.19243	2018	1
BRA	Brazil	Bahia	Amaranthaceae	unidentified	−9.99094	−38.63050	2018	1
BRA	Brazil	Bahia	Portulacaceae	*Portulaca* sp.	−9.99094	−38.63050	2018	4
Non‐native range distribution								
PRC	Puerto Rico	CMuertos	Cactaceae	*Stenocereus fimbriatus*	17.89625	−66.52170	2016	5
PRC	Puerto Rico	Main Island	Cactaceae	*Pilosocereus royenii*	17.97880	−67.16903	2016	6
PRC	Puerto Rico	Main Island	Cactaceae	*Pilosocereus royenii*	17.95911	−66.86148	2016	8
PRC	Puerto Rico	Main Island	Cactaceae	*Hylocereus trigonus*	17.95223	−66.38332	2016	4
PRC	Puerto Rico	Main Island	Cactaceae	*Hylocereus trigonus*	17.95302	−66.38361	2018	5
PRC	Puerto Rico	Main Island	Cactaceae	*Pilosocereus royenii*	17.95223	−66.38332	2018	7
PRC	Puerto Rico	Main Island	Cactaceae	*Melocactus intortus*	17.96014	−66.86125	2018	5
PRC	Puerto Rico	Main Island	Cactaceae	*Stenocereus fimbriatus*	17.97877	−67.16907	2018	2
PRA	Puerto Rico	Vieques	Amaranthaceae	*Achirantes aspera*	18.09362	−65.55121	2019	5
PRA	Puerto Rico	Culebrita	Amaranthaceae	*Alternanthera crucis*	18.32333	−65.22775	2018	6
PRA	Puerto Rico	Main Island	Amaranthaceae	*Gomphrena serrata*	17.93703	−66.18419	2018	4
PRA	Puerto Rico	Culebra	Portulacaceae	*Portulaca* sp.	18.32302	−65.22784	2019	3
PRA	Puerto Rico	Vieques	Amaranthaceae	*Achirantes aspera*	18.09653	−65.52499	2018	6
PRA	Puerto Rico	Main Island	Portulacaceae	*Portulaca teretrifolia*	18.01182	−66.25125	2018	4
PRA	Puerto Rico	Vieques	Portulacaceae	*Portulaca* sp.	18.09653	−65.52499	2018	2
PRA	Puerto Rico	Vieques	Portulacaceae	*Portulaca* sp.	18.09653	−65.52499	2018	5
USA	United States	Florida	Amaranthaceae	*Alternanthera ficoidea*	28.63787	−81.54858	2017	6
USA	United States	Florida	Amaranthaceae	*Froelichia floridana*	26.56874	−80.07243	2017	5
USA	United States	California	Cactaceae	Cactaceae	34.07362	−118.40035	2019	5
ARC	Australia	Queensland	Cactaceae	*Harrisia martinii*	−27.59750	151.77556	2016	5

Genomic DNA was extracted using entire bodies of adult female mealybugs using Qiagen DNeasy Blood & Tissue Kit according to manufacturer's instructions, adding 2 µl of RNAse A after the lysis step. DNA was quantified using Qubit 2.0 Fluorometer (Life Technologies), and quality was assessed in a NanoDrop ND‐1000 spectrophotometer (NanoDrop Technologies Inc.).

### 2.2 NextRAD sequencing

Genomic DNA was converted into nextRAD genotyping‐by‐sequencing libraries (SNPsaurus, LLC) as in Russello, Waterhouse, Etter, and Johnson, (2015). Genomic DNAs were first fragmented with Nextera DNA Flex reagent (Illumina, Inc.), which also ligates short adapter sequences to the ends of the fragments. The Nextera reaction was scaled at a 1/5th reaction, and 5 ng of genomic DNA was used due to the presence of inhibitors in the samples. Fragmented DNAs were then PCR amplified for 26 cycles at 73ºC, with one of the primers matching the adapter and extending 9 nucleotides into the genomic DNA with the selective sequence GTGTAGAGC. Thus, only fragments starting with a sequence that matched the selective sequence of the primer were efficiently amplified. NextRAD libraries were sequenced in a 150 bp single‐end reads mode in a lane of a HiSeq 4000 (SNPSaurus at University of Oregon). Raw sequence data are available at the National Centre for Biotechnology Information (NCBI), under the BioProject accession number PRJNA593002.

### 2.3 SNP calling and genomic data filtering

The genotyping analysis used custom scripts (SNPsaurus, LLC) that trimmed the reads using bbduk (BBMap, http://sourceforge.net/projects/bbmap/) (Bushnell, 2017) to remove Nextera adaptors and low‐quality sequences. A reference draft genome was built using DNA from a single male mealybug from Catamarca, Argentina (Table 1). By means of this procedure, we were able to obtain a clean reference since males do not feed as adults, allowing us to identify reads of bacteria, parasitoids or food scraps present in females’ sequences. To build the reference genome, 150 bp paired‐end reads were sequenced in a lane of a HiSeq 4000 (SNPSaurus at University of Oregon). Illumina paired‐end sequences were then trimmed for Nextera adapters using bbduk (BBMap,sourceforge.net/projects/bbmap) (Bushnell, 2017). The assembly was done using abyss‐pe (Jackman et al., 2017). Assembled contigs shorter than 250 bp were removed and then aligned using blastn to the NCBI nt database. Blast hits to bacterial species were removed. Reference draft genome is available as FuEDEI_HPun_1.1.fa at the NCBI under accession number: JAAOIU000000000. Cleaned reads were then mapped to the reference mealybug draft genome with an alignment identity threshold of 0.95 using bbmap (BBMap tools). Genotype calling was done by using callvariants (BBMap tools). The Variant Calling File generated was filtered to remove individuals with more than 10% missing data, sites with more than 20% missing genotypes, and loci with minor allele frequency (MAF) lower than 0.03 using VCFtools v1.15 (Danecek et al., 2011). We randomly selected one SNP per contig to minimize linkage disequilibrium (LD) and to ensure the independence of the SNPs employed in the next analyses. We also excluded from our dataset SNPs with more than two allele variants and indels. We used BayeScan to test for outlier SNPs. Fit to Hardy–Weinberg expectations (HWE) of variant frequencies for each locus within populations was tested using the exact test implemented in dDocent (Puritz, Hollenbeck, & Gold, 2014).

### 2.4 Mitochondrial gene amplification and sequencing

A fragment of the mitochondrial gene encoding for the Cytochrome oxidase subunit I (*COI*) was amplified using primers C1‐J‐2183 Jerry (Simon et al., 1994) and C1‐N‐2568 BEN3R (Brady, Gadau, & Ward, 2000). PCR reactions were performed following Poveda‐Martínez et al. (2019). PCR products were checked in 1% agarose gels and purified using the QIAquick PCR Purification Kit (Qiagen). Finally, both strands of the amplicons were sequenced using Sanger methodology at Macrogen Inc. The mtDNA haplotypes found in this study from Argentina, Brazil, Paraguay, Puerto Rico, and the United States were deposited in GenBank under access numbers: MT138921‐MT138931. Additional *COI* haplotypes from Argentina detected previously in Poveda‐Martínez et al. (2019), were retrieved from GenBank (MN013440 ‐ MN013554).

### 2.5 Population genomics, clustering, and phylogeographic analyses

Based on SNPs datasets, observed heterozygosity (*H*_o_) and allele richness (Ar) were calculated with R package *hierfstat* (Goudet, 2005). Expected heterozygosity (*H*_e_), global differentiation estimates (*G*_st_), and pairwise *F*_st_ estimates between populations from native and non‐native ranges were calculated using *mmod* and *Adegenet* (Jombart, 2008) packages available in R (www.r‐project.org). Populations were defined by sampling location and host plant collection site; however, we decided to pool samples collected in Argentina, Paraguay, and Australia due to low sample size after SNPs filtering. This decision is justified since Australian mealybugs have been shown to be part of the same clade with mealybugs from Argentina feeding on Cactaceae (Poveda‐Martínez et al., 2019) and are known to be derived from introductions of mealybugs collected in Argentina on Cactaceae. Sample sizes were balanced with the number of informative loci recovered. In this context, it is important to recall that even small sample sizes can accurately account for genetic divergence when the number of SNPs is >1,500 (Nazareno, Bemmels, Dick, & Lohmann, 2017). To assess population structure, we employed two approaches. First, we used sNMF (Frichot, Mathieu, Trouillon, Bouchard, & François, 2014) as implemented in R using the LEA package (Frichot & François, 2015) to assign samples to genetic clusters. sNMF estimates individual ancestry coefficients utilizing the same likelihood model underlying STRUCTURE (Pritchard, Stephens, & Donnelly, 2000) and ADMIXTURE (Alexander, Novembre, & Lange, 2009), but uses nonnegative matrix factorization and least squares optimization to accommodate genome scale datasets. Ancestry coefficients were calculated by sNMF for *K* values ranging from 1 to 10, with 100 repetitions for each value of *K*, and the optimal *K* value was assessed using the cross‐entropy criterion based on the prediction of masked genotypes to evaluate the error of ancestry estimation. The second approach used to assess population structure was a discriminant analysis of principal components DAPC (Jombart, Devillard, & Balloux, 2010) as implemented in R in *adegenet* package, using *K* means clustering to identify the optimal number of clusters for *K* values ranging from 1 to 10, and estimating individual admixture coefficients. We then calculated Bayesian information criterion scores (BIC) to identify the K value with the best fit. Additional pairwise *F*_st_ estimates between groups identified in prior clustering analyses were performed using the SNPs dataset (142 individuals, 1,707 SNPs).

Mitochondrial DNA sequences were aligned using Clustal W implemented in MEGA v7 (Kumar, Stecher, & Tamura, 2016) and used to build a haplotype Network using median Joining algorithm (Bandelt, Forster, & Röhl, 1999) implemented in PopArt v1.7.1 (Leigh & Bryant, 2015). Additionally, we calculated nucleotide diversity (π), haplotype diversity (*H*_d_), and number of haplotypes (H). Pairwise *F*_st_ estimates between populations (as defined earlier) were calculated using DNAsp v6 (Rozas et al., 2017).

### 2.6 Influence of host plants and geographic distance on genetic divergence

We analyzed the influence of geographic distance and host plants on genetic divergence among *H. pungens* complex populations from the native range. Non‐native populations in these analyses were excluded because samples collected in these locations reached the areas by human mediated dispersal and would distort the analyses. We used a combination of correlation tests and multiple regression analyses of distance matrices to analyze the importance of geographic distance and host plant use on genetic divergence.

In these analyses, we used pairwise *F*_st_ linearized values at population level based either on nuclear SNPs or mtDNA datasets to construct the matrix of genetic distances. To construct the geographic distance matrix, we used the coordinates (latitude and longitude) of each population (Table 1). Host plant distance matrix was built considering host plant families where mealybugs were collected: Cactaceae, Portulacaceae, and Amaranthaceae. Thus, we constructed a binary matrix with three categories (the host plant families) considering patterns of host plant use.

To analyze the influence of geographic distance and host plant use on genetic divergence, we employed two Mantel tests (Mantel, 1967), first considering geographic and genetic matrices and second host plant and genetic distance matrices. We then conducted a partial Mantel test (Smouse, Long, & Sokal, 1986) to evaluate the influence of host plants on genetic divergence, using pairwise *F*_st_ and host plant distance matrix, while controlling for geographic distance (Legendre & Fortin, 2010). For all correlation analyses, we used the R package *vegan* v2.4.4 (Oksanen et al., 2007) and 9,999 permutations to assess statistical significance. We further tested the influence of geographic distance and host plant use on genetic divergence by performing matrix regressions using the function MRM from the package *ecodist* v2.0.1 (Goslee & Urban, 2007) and ran 9,999 permutation to assess the significance of regression coefficients.

### 2.7 Species tree reconstruction and species delimitation analyses

For SNPs and mtDNA datasets, maximum likelihood (ML) and Bayesian inference (BI) approaches were performed. ML for both datasets was implemented in IQ‐TREE v1.6.10 (Nguyen, Schmidt, Von Haeseler, & Minh, 2015) using CIPRES (Miller, Pfeiffer, & Schwartz, 2010) with 10,000 ultrafast bootstrap (UFboot) iterations. ModelFinder estimated GTR as the best‐fitted model according to Akaike information criterion (AIC) for SNPs dataset and GTR + Γ + I for mtDNA dataset. BI reconstruction was assessed using MrBayes v3.2.6 (Ronquist et al., 2012), employing the entire matrix of 1,707 SNPs and the mtDNA haplotypes following the same fit‐model implemented in ML analyses. For each dataset, two independent runs were performed with four Markov Chain Monte Carlo (MCMC) for each run for 50 million generations, sampling every 1,000 generations. The first 25% of the runs were removed as burn‐in, and stability and sufficient mixing of parameters (ESS > 1,000 for SNPs and ESS > 200, for mtDNA) were checked using Tracer v1.6 (Rambaut, Suchard, Xie, & Drummond, 2014). UFboot values and posterior probabilities (PP) resulted from ML and BI, respectively, were plotted on each node of the reconstructed tree of each dataset. Additionally, based on SNPs dataset, a species tree was estimated using the coalescent‐based methods, SNAPP (Bryant, Bouckaert, Felsenstein, Rosenberg, & RoyChoudhury, 2012) implemented in Beast v2.5.2 (Bouckaert et al., 2014). In this case, we could only include two individuals, selecting individuals with the lowest numbers of missing data, from each one of the groups defined in clustering analyses (see the previous section) as a result of the high computational demand of the method. Due to the reduction of the number of individuals, some sites could become monomorphic either because of the reference or the alternative allele, and some sites might also have had no data left for one or more of the sampled localities. Therefore, we decided to filter out monomorphic sites and sites with no data for one or more populations with bcftools v1.7 (Li et al., 2009), resulting in a new matrix of 1,679 SNPs. We used the default model parameters in SNAPP for U and V equal to one, and we ran the analysis for 10,000,000 MCMC generations, sampling every 1,000 generations. Stationarity and convergence of data were checked using Tracer v1.6 (Rambaut et al., 2014). The complete set of trees were visualized in Densitree v2.2.5 (Bouckaert & Heled, 2014), removing the first 10% of the trees as burn‐in. Finally, we generated a maximum clade credibility tree using TreeAnnotator v1.7.5 (Drummond, Suchard, Xie, & Rambaut, 2012) to access the posterior probabilities of the resulted topology.

Bayes factor species delimitation (BFD*) (Leaché, Fujita, Minin, & Bouckaert, 2014) analysis, based on the SNPs dataset, was performed using the SNAPP and *Path Sampler* packages included in Beast v2.5.2 (Bouckaert et al., 2014). We estimated and compared the marginal likelihood estimates (MLE) for two models of species limits. Model 1 considered *H. pungens* as a complex of the following five species according to the results obtained with sNMF, DAPC, and the estimated best phylogenetic tree: (a) *H. pungen*s sensu stricto (*Hypogeococcus* populations collected on Amaranthaceae in Argentina); (b) mealybugs feeding on Cactaceae from Argentina–Paraguay–Australia; (c) mealybugs feeding on Cactaceae from southeastern Brazil and Puerto Rico; (d) mealybugs feeding on Amaranthaceae and Portulacaceae from northeastern Brazil, Puerto Rico, and the United States; and (e) mealybugs feeding on Amaranthaceae from southeastern Brazil. Model 2 considered *H. pungens* as a complex comprising four species according to the major clades found with mtDNA: the same groups as in Model 1 with the difference that *H. pungen*s sensu stricto and mealybugs feeding on Amaranthaceae and Portulacaceae from northeastern Brazil, Puerto Rico, and the United States appear as only one group. The MLE for each model was calculated using the same mutation rate as above, considering 12 steps with an MCMC length of 200,000 generations, a preburning of 20,000 and discarding the first 10% as burning. The MLEs for each model were used to calculate the Bayes factor (BF) test statistics 2*MLE(model1*)*‐MLE*(*model2*) (Kass & Raftery, 1995).

Finally, two single locus methods of species delimitation were run based on mtDNA, the Generalized Mixed Yule Coalescent (GYMC) (Pons et al., 2006) and Bayesian Poisson tree processes (bPTP) (Zhang, Kapli, Pavlidis, & Stamatakis, 2013). For the GYMC analysis, we first calculated an ultrametric tree in Beast v2.5.2 (Bouckaert et al., 2014). We used GTR + Γ + I model with an uncorrelated relaxed clock and constant size tree prior. MCMC was set to 10,000,000 generations, sampling every 1,000th generation. The maximum credibility tree was generated in TreeAnnotator v1.7.5 (Drummond et al., 2012). The GYMC analysis was performed with the *SPLITS* package (Ezard, Fujisawa, & Barraclough, 2009) in R, using the ultrametric tree inferred by Beast. The bPTP analyses were performed in the bPTP server (http://species.h‐its.org/) using the ultrametric tree in nexus format, with default values and using 200,000 MCMC generations.

## 3 RESULTS

### 3.1 NextRAD and mitochondrial data

The nextRAD dataset consisted, on average, of 1.6 million single‐end reads per individual, with an average read length of 123 bp. The final assembled genome contained 250,856 fragments with a total length of 240.6 Mb. Considering a set consisting of 173 samples, we identified 14,446 loci. However, we had to remove 32 individuals from further analyses because of high numbers of missing data (>20% missing genotypes) following recommendations for population genomic analyses (Arnold, Corbett‐Detig, Hartl, & Bomblies, 2013). From the remaining 141 individuals, we recovered a total of 5,383 loci. After filtering loci that were present in at least 90% of the individuals, we ended up with a final dataset containing 1,707 SNPs (average coverage 78X) used for further analyses. Numbers of loci removed in each filtering step are detailed in Table 2. Since the 10% of missing data criterium used for filtering the SNPs dataset is the strictest threshold, we evaluated other filtering thresholds that yielded essentially the same results in population genetic structure and species delimitation analyses.

**TABLE 2:** Number of loci identified after each quality filtering step was conducted on the final sample of 141 *Hypogeococcus pungens* species complex individuals collected in native and non‐native areas

Filtering steps	SNPs retained
Initial after variant calling	14,446
Genotyped successfully in 90% of individuals^a^	5,383
MAF (<0.01)	5,070
No indels	4,519
Random select one SNP per contig	1,733
HWE by population (*p* < 0.05)	1,707
*F*_st_ outliers (CI 5%)	0
Total SNPs used in analyses	1,707

Abbreviations: HWE, Hardy–Weinberg Equilibrium; MAF, Minor allele frequency.

^a^ 32 individuals also were removed due to high levels of missing data.

The mtDNA dataset consisted of 233 sequences, 143 collected in the native range and 90 in the non‐native range. Additionally, we included *COI* sequences of five individuals from California, United States on Cactaceae, but SNPs data at this location could not be generated. The length of the amplified *COI* fragment was 413 bp, with 371 conserved (89.83%) and 42 (10.16%) variable sites, distributed in 27 haplotypes. The mtDNA sequences are available in GenBank under accession numbers MT138921 ‐ MT138931.

### 3.2 Genomic diversity and differentiation in the *H. pungens* species complex

Both SNPs and mtDNA datasets revealed intermediate levels of genetic diversity but great levels of differentiation among populations. Overall nuclear and mitochondrial genetic diversity were high across the native range and low in the non‐native range (Table 3). Based on SNPs data, total mean expected heterozygosity (*H*_e_ = 0.061) was three times greater than observed heterozygosity (mean *H*_o_ = 0.029), probably as a consequence of differentiation among populations (see below). Expected heterozygosity varied greatly among populations ranging from 0.267 in the native range (mean *H*_e_ = 0.102) to as low as 0.003 in the non‐native area (mean *H*_e_ = 0.061). Allele richness was 20% higher in the native range (mean Ar = 1.240) than in recently invaded areas (mean Ar = 1.020), and no private alleles were detected.

**TABLE 3:** Measures of genetic diversity in *Hypogeococcus pungens* species complex collected in the insects’ native and non‐native ranges based on datasets consisting of 1,707 neutral SNPs and mtDNA

Population code	nextRAD				mtDNA			
	*n*	*H*_e_	*H*_o_	Ar	*n*	*H*_d_	π	*H*
Native range								
ARA	8	0.0961	0.1259	1.2747	63	0.861	0.0049	11
ARC	24	0.0170	0.0111	1.0496	43	0.787	0.0114	7
BRA	15	0.2674	0.0183	1.5670	19	0.807	0.0152	7
BRC	20	0.0272	0.0234	1.0688	18	0.523	0.0024	2
	Mean	0.1019	0.0450	1.2400	Mean	0.744	0.0085	Total = 27
Non‐native range								
PRC	34	0.0125	0.0152	1.0322	57	0	0	1
PRA	30	0.0075	0.0088	1.0212	23	0.522	0.0012	2
USA	11	0.0028	0.0044	1.0088	10	0	0	1
	Mean	0.0076	0.0094	1.0207	Mean	0.174	0.0004	Total = 3
	Total mean	0.0615	0.0296	1.1460	Total mean	0.459	0.0044	

Note

*n*: Sample size; *H*_e_: expected heterozygosity; *H*_o_: observed heterozygosity; Ar: Allelic richness; *H*_d_: Haplotype diversity; π: Nucleotide diversity; *H*: number of haplotypes.

Abbreviations: ARA, Argentina mealybugs feeding on Amaranthaceae; ARC, Argentina, Australia and Paraguay mealybugs feeding on Cactaceae; BRA, Brazil mealybugs feeding on Amaranthaceae; BRC, Brazil mealybugs feeding on Cactaceae.

The analysis of pairwise *F*_st_ values estimated for the SNPs dataset, considering the a priori defined populations, sampling area, and host plant family, showed great global genetic differentiation (*G*_st_ = 0.908). In the native range, *F*_st_ estimates ranged from 0.602 for the comparison between BRC (Brazilian mealybugs feeding on Cactaceae) and BRA (Brazilian mealybugs feeding on Amaranthaceae and Portulacaceae) to 0.827 in the comparison between ARA (Argentina mealybugs feeding on Amaranthaceae) and ARC (Argentina, Paraguay, and Australia mealybugs feeding on Cactaceae). Genetic differentiation (*F*_st_) in the non‐native area varied from values as low as 0.078 between PRA (Puerto Rican mealybugs feeding on Amaranthaceae or Portulacaceae) and USA (United States mealybugs feeding on Amaranthaceae) to as high as 0.969 between PRA and PRC (the Puerto Rican cactus pest) (Table S1).

**FIGURE 2:**
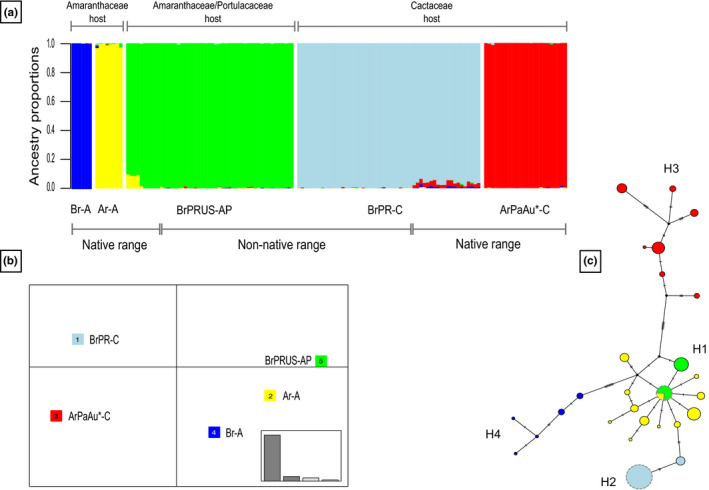
Clustering analyses using sNMF (a) and DAPC (b) methods based on 1,707 SNPs from 141 *Hypogeococcus pungens* species complex specimens and neighbor joining network based on mtDNA data (c). Visual representation of five clusters (*K* = 5) along with membership probability of each individual to the corresponding cluster. Color codes in both clustering analyses and in the neighbor joining network are the same: yellow: Ar‐A: specimens from Argentina feeding on Amaranthaceae; green: BrPRUS‐AP: specimens from northern Brazil, Puerto Rico, and the United States feeding on Amaranthaceae and/or Portulacaceae; red: ArPaAu‐C: specimens from Argentina, Paraguay, and Australia feeding on Cactaceae; light blue: BrPR‐C: specimens from southern Brazil and Puerto Rico feeding on Cactaceae and blue: Br‐A: specimens from southern Brazil feeding on Amaranthaceae

Clustering analyses also revealed strong population structuring in the *H. pungens* species complex, not necessarily associated with site location. Indeed, sNMF and DAPC analyses suggested that the *H. pungens* species complex comprises five ancestral populations/ clusters (*K*) based on cross‐entropy and BIC values (Figure 2a,b, respectively). The first cluster corresponds to what was previously called *H. pungens* sensu stricto (Ar‐A) (Poveda‐Martínez et al., 2019) that included Argentina (Ar) mealybugs feeding on Amaranthaceae (A). The second cluster included Cactaceae (C) feeding mealybugs native from Argentina, Paraguay (Pa), and mealybugs collected in Australia (Au) (ArPaAu‐C). The third cluster included populations of Cactaceae‐feeding mealybugs from southern Brazil (Br) and the Puerto Rican (PR) cactus pest (BrPR‐C), whereas the fourth cluster encompassed mealybugs feeding on Amaranthaceae and/or Portulacaceae (AP) species from northeastern Brazil along with populations from Puerto Rico and the United States (US) (BrPRUS‐AP). The last cluster included mealybugs collected on Amaranthaceae in southeastern Brazil (Br‐A). Admixture coefficients suggested no admixture between clusters and strong population genetic structure. Pairwise *F*_st_ values considering the five clusters described above revealed strong genetic differentiation, *F*_st_ estimates ranged from 0.567 in the comparison between Ar‐A, and BrPRUS‐AP to 0.945 in the comparison between BrPRUS‐AP and Br‐A (Table S2).

Concerning the mtDNA dataset (Table 3), a total of 27 haplotypes were found in native and non‐native ranges. All haplotypes were present in the native range, and 18 haplotypes were detected in mealybug samples collected on Amaranthaceae and/or Portulacaceae hosts and 9 on Cactaceae hosts (mean *H*_d_ = 0.744). As expected, the number of haplotypes was substantially lower in non‐native sites, where only three haplotypes were found (mean *H*_d_ = 0.174), two in samples collected on Amaranthaceae and/or Portulacaceae hosts and only one on Cactaceae hosts.

Four haplogroups, separated by at least two substitutions, can be recognized based on mtDNA data, each one characterized by the type of host plant (Amaranthaceae and/or Portulacaceae, or Cactaceae) and geographic origin (Figure 2c). The first haplogroup (H1), which contained the most frequent haplotype (haplotype MT138927) and has a star‐like shape, is shared by Amaranthaceae‐feeding mealybugs collected in Argentina and specimens from Brazil, Puerto Rico, and United States (Florida) feeding on Amaranthaceae and/or Portulacaceae. The second haplogroup (H2), which includes two haplotypes, both from mealybugs using Cactaceae as host plants, one (haplotype MT138924) is shared by the mealybugs from southeastern Brazil, Puerto Rico, and United States (California), and the other (haplotype MT138923) only found in southeastern Brazil. The remaining two haplogroups are highly differentiated from H1 and H2. H3 included Cactaceae‐feeding mealybugs collected in Argentina, Australia, and Paraguay and H4 Amaranthaceae‐feeding mealybugs from southeastern Brazil.

### 3.3 Influence of host plant use and geographic distance in genetic variation

Analyses of the influence of host plants and geographic distance on genetic variation in the *H. pungens* species complex showed that host plant use was a better predictor of genetic distance than geographic distance (Table 4). Correlation tests between host plant use and genetic distance matrices and between geographic and genetic distance matrices using the nuclear SNPs dataset were significant (*r* = 0.5254; *p* = 0.0017 and *r = *0.4; *p* = 0.0128, respectively). In addition, the partial Mantel test indicated that the correlation between host plant distance and genetic distance matrices remained significant when controlling for geographical distance (*r* = 0.6133; *p* = 0.0001). Likewise, the results of regressing the matrix of genetic distances on both host plant and geographic distances showed that both factors significantly affected genetic differentiation among sampling localities for nuclear SNPs (host plant coefficient = 0.28497, *p* = 0.0001; geographic coefficient = 0.00709, *p* = 0.0001). Host plant and geographic distance together explained 47.6% of genetic variation. In contrast, correlation and multiple regression tests based on mtDNA distance matrix on geographic and host plant distances yielded nonsignificant results (Table 4).

**TABLE 4:** Effect of host plant use and geography on genetic variation of *Hypogeococcus pungens* species complex populations based on SNPs and mtDNA datasets

	SNPs		mtDNA	
	*R*	*p*	*R*	*p*
Correlation matrices by Mantel test				
Genetic distance and geographical distance	**0.4000**	**0.0128**	−0.0524	0.5859
Genetic distance and host plant use	**0.5254**	**0.0017**	0.2625	0.0648
Genetic distance + host plant – Geography^a^	**0.6133**	**0.0001**	0.2631	0.0664

	SNPs		mtDNA	
	Coefficient	*p*	Coefficient	*p*
Multiple Regression matrix by MRM				
Intercept	0.52924	0.9808	0.74032	0.7671
Geography	0.00709	**0.0001**	−0.0012	0.7651
Host plant use	**0.28497**	**0.0001**	0.1355	0.0999
	Model* R* ^2^	**0.4759**	Model* R* ^2^	0.0717
	*p*	**0.0001**	*p*	0.2622

^a^ Correlation analysis was performed using the partial Mantel test. Significantly different tests are designated by bold font.

### 3.4 Species tree reconstruction and species delimitation analysis

**FIGURE 3:**
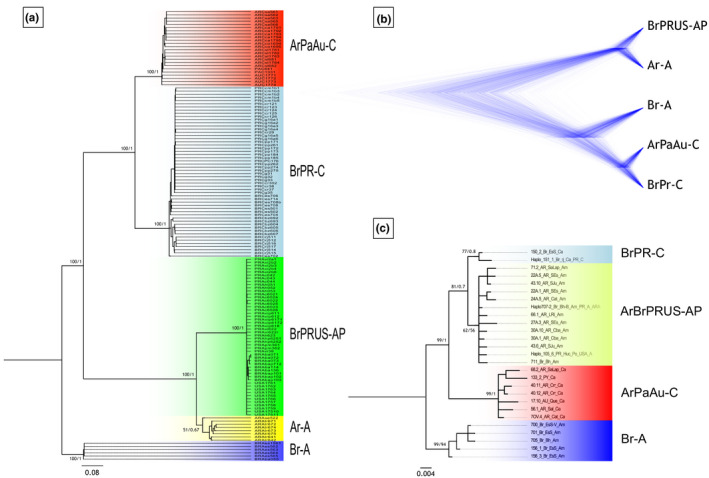
Phylogenetic hypotheses based on SNPs and mtDNA for *Hypogeococcus pungens* species complex. Phylogenetic tree inferred by ML and BI based on 1,707 SNPs (a), species tree reconstructed using coalescent method in SNAPP based on the dataset of 1,679 SNPs (b), phylogenetic tree inferred by ML and BI based on mtDNA haplotypes (c). Mealybugs feeding on Cactaceae host plant: clade ArPaAu‐C, mealybugs from Argentina, Paraguay, and Australia, and mealybugs from southeastern Brazil and Puerto Rico (clade BrPR‐C). Mealybugs feeding on Amaranthaceae and Portulacaceae host plant: Br‐A, mealybugs from southeastern Brazil; clade Ar‐A, *H. pungens* sensu stricto; clade BrPRUS‐AP, mealybugs from northeastern Brazil, Puerto Rico and the United States

Both ML and BI phylogenetic analyses and the species tree inferred by SNAPP, all of them based on the SNPs dataset, revealed five major well supported clades (Figure 3a,b). The first and second clades, both feeding on Amaranthaceae and/or Portulacaceae, appear closely related, the first encompassing *H. pungens* sensu stricto (clade Ar‐A) and the second mealybugs from northeastern Brazil, Puerto Rico, and United States (Florida) (clade BrPRUS‐AP), respectively. The third and fourth clades comprised cactus feeding mealybugs: the third from Argentina, Paraguay, and Australia (clade ArPaAu‐C) and the fourth from southern Brazil and Puerto Rico (clade BrPR‐C). The fifth clade was an independent branch formed by Amaranthaceae‐feeding mealybugs collected in southeastern Brazil (clade Br‐A) (Figure 3a,b).

The phylogenetic tree obtained with the mtDNA dataset showed contrasting results with respect to nuclear SNPs (Figure 3c). Four major clades were observed in ML and BI trees, one encompassing *H. pungens* sensu stricto with populations from northeastern Brazil, Puerto Rico, and the United States (Florida) feeding on Amaranthaceae and/or Portulacaceae (clade ArBrPRUS‐AP). The clade including cactus feeding mealybugs from southeast Brazil and Puerto Rico (clade BrPR‐C) appears as the sister clade of ArBrPRUS‐AP. The third clade included populations from Argentina, Paraguay, and Australia feeding on Cactaceae (clade ArPaAu‐C) that appeared close to Amaranthaceae‐feeding mealybugs from southeastern Brazil (clade Br‐A) (Figure 3c).

Species delimitation analysis based on the BFD* method using SNPs data supported a model in which *H. pungens* was a complex of five species (Model 1, Table S3), supporting the picture depicted by the phylogenetic tree and species tree produced with SNPs data (Figure 3a,b). This means that the clade which included *H. pungens* sensu stricto (Ar‐A) and the clade comprising mealybugs from northeastern Brazil, Puerto Rico, and the United States (Florida) feeding on Amaranthaceae and/or Portulacaceae (BrPRUS‐AP), as well as the clade of Amaranthaceae feeders from southeastern Brazil (Br‐A), may be considered as three separate species. Moreover, the latter appeared as the sister clade of the two clades of cactus feeders, one from Argentina, Paraguay, and Australia (clade ArPaAu‐C) and the other from southeastern Brazil and Puerto Rico (clade BrPR‐C).

GYMC and bPTP, the single locus species delimitation methods based on mtDNA, recovered six (GYMC) and 10 (bTPT) groups (Figure S1). In both cases, GYMC and bPTP delimited four of the five groups identified with SNPs data. These single locus methods did not consider the fifth group that consisted of *H. pungens* sensu stricto and the population from northeastern Brazil, Puerto Rico, and the United States (Florida) on Amaranthaceae and/or Portulacaceae. Both methods delimited three (GYMC) and four (bPTP) additional groups in the clade conformed by mealybugs from Argentina, Paraguay, and Australia feeding on Cactaceae. Two additional splits were considered by bPTP, the first in *H. pungens* sensu stricto and the second one in mealybugs from southeastern Brazil on Amaranthaceae (Figure S1).

## 4 DISCUSSION

Our study confirmed that *H. pungens*, commonly called the *Harrisia* cactus mealybug (HCM), is not a single polyphagous species, but a species complex consisting of at least five species. Each member of the complex was associated with particular hosts, indicating a high degree of specificity at the host plant family level. Our genomic survey allowed the identification of the source population from which the Puerto Rico cactus pest was derived. This is an important achievement since it will help to design reliable strategies for classical biological control using natural enemies, such as specialized parasitoids, associated with the pest in its native range.

### 4.1 *Hypogeococcus pungens* species complex and host plant use

The specimens used to describe *H. pungens* were originally collected on an Amaranthaceae host (Granara de Willink, 1981). Further collections extended its host range to the Cactaceae, and Portulacaceae families (Ben‐Dov, 1994; Claps & de Haro, 2001; Zimmermann et al., 2010), suggesting that *H. pungens* was a polyphagous species. However, our population genomics approach pointed to a clear separation between the mealybugs associated with Amaranthaceae or Cactaceae and others that indiscriminately use Amaranthaceae and Portulacaceae species as hosts. These results confirmed that *H. pungens* is not a polyphagous species, but a species complex composed of cryptic species associated with different host plants as feeding resources. Population genomics, clustering, and species delimitation analyses based on genome‐wide SNPs and the mitochondrial gene *COI* datasets revealed deep genetic divergence among populations formerly considered as part of *H. pungens*.

These results agree with previous studies that revealed a deep genetic divergence, asymmetrical prezygotic and postzygotic reproductive isolation between the mealybug populations associated with different hosts from Argentina and differential preference and performance on alternative hosts (Aguirre et al., 2016; Poveda‐Martínez et al., 2019). The extension of our sampling effort to a wider geographic area throughout the native and non‐native ranges, along with the use of a substantially large number of nuclear markers, allowed the confirmation of the results reported for the Argentine *Hypogeococcus* mealybugs (Poveda‐Martínez et al., 2019) and new insights of the evolutionary history of *H. pungens* sensu lato. In effect, our present results support the hypothesis that genetic differentiation throughout the entire range in South America has mainly been driven by divergent selection imposed by alternate host plants rather than by isolation through distance. In fact, the results of Mantel correlation analyses point to a relationship (not necessarily causal) between genetic, geographic, and host plant distance matrices. Even though there has been a vibrant debate regarding the proper (and improper) use of Mantel tests for population genetic and phylogenetic data (Guillot & Rousset, 2013; Harmon & Glor, 2010; Legendre & Fortin, 2010; Legendre, Fortin, & Borcard, 2015), it should be recalled that our conclusions are also based on the results of a multiple regression analysis that revealed that host plant use (accounting for more than 48% of total variance) is a better predictor of genetic divergence among populations (Table 4).

In this context, our results pointed to host plant use as an important driver of cryptic divergence in an a priori presumed polyphagous insects. Cryptic divergence has been recurrently observed in the form of host‐associated species that, very likely, diverged as the result of adaptation to alternate host plants with little morphological divergence in several insect species (Bagley, Sousa, Niemiller, & Linnen, 2017; Driscoe et al., 2019; Forbes et al., 2017; Matsubayashi, Kahono, & Katakura, 2011; Zhang, Bass, Fernández, & Sharanowski, 2018).

Genetic divergence between the cactus feeding mealybugs native to Argentina and Paraguay and those introduced to Australia from Argentina (ArPaAu‐C) and cactus feeding mealybugs from southeastern Brazil (BrPr‐C) exceeded that which would be expected for conspecific populations, as indicated by our species delimitation analyses. In this context, it is worth mentioning that besides the large geographic distance separating populations of these clades and the fact that both feed on Cactaceae, these two putative species fed on cactus that belong to different genera. Mealybugs of the ArAuPa‐C clade fed on species of the genera *Cleistocactus* and *Harrisia*, whereas BrPr‐C thrived on species of other genera like *Pilosocereus* for native Brazilian mealybugs, and various genera for non‐native Puerto Rican pest mealybugs (Table 1). It may be argued that divergent selection on alternate hosts and allopatry appeared as the more likely drivers of divergence. As a matter of fact, there is evidence that divergent selection caused by alternate hosts acting on ancestral fragmented populations is sufficient to produce genetic divergence and incidental speciation (Doellman et al., 2019; Duque‐Gamboa et al., 2018; Nosil et al., 2012).

Our results also elucidated a certain degree of genetic heterogeneity within the ArAuPa‐C clade, which included cactus feeders sampled in Argentina, Paraguay, and those introduced in Australia for biological control of cactus weeds. Both mtDNA and nuclear SNPs revealed a certain degree of genetic structuring within this clade. Several well‐differentiated mtDNA haplotypes were recorded at different locations of this widespread clade, likely as the result of geographic isolation (Figure 2c). Also, the phylogenetic tree based on nuclear SNPs showed internal subclades concordant with the results depicted in the *COI* network. An evaluation of the external morphology of specimens collected on cacti in the same localities sampled for the genomic survey in Argentina revealed subtle morphological variation (Lucía Claps, University of Tucumán, Argentina, personal communication), suggesting that genetic heterogeneity in this clade may be indicative of incipient speciation rather than within species heterogeneity. These results also helped to end the debate of the controversial origin of the mealybugs used in the biological control program in Australia (and in South Africa) (Hamon, 1984; Tomley & McFadyen, 1984; Williams, 1973). Indeed, Australian *Hypogeococcus* mealybugs were genetically close to the cactus feeding samples collected in northwestern Argentina and Paraguay, but not, as suggested by other authors, to *Hypogeococcus festerianus* Lizer & Trelles, another cactophagous species inhabiting central‐western Argentina, or *H. pungens* (Julien & Griffiths, 1999; McFadyen, 2012; Zimmermann et al., 2010).

Overall, our data provided evidence of two factors that influenced at least 48% (Table 4) of the genetic divergence between the mealybugs considered as part of the *H. pungens* species complex. Host plant associations seemed to be the primary force influencing genetic divergence, followed by the limited gene flow induced by isolation by distance. Still, these results should be interpreted with caution since other factors could also affect the distribution of genetic variation that remained unexplained. Local adaptation to different environmental conditions and/or ecological interactions with natural enemies or competitors may impose varying selective pressures in different geographic locations. A recent survey of parasitoids and hyperparasitoids associated with *Hypogeococcus* mealybugs in South America identified *Leptomastidea hypogeococci* Triapitsyn (Hymenoptera: Encyrtidae) as a widespread primary parasitoid, able to attack all members of the *H. pungens* complex, whereas *Anagyrus cachamai* Triapitsyn, Logarzo & Aguirre, and *A. lapachosus* Triapitsyn, Aguirre & Logarzo (also encyrtid primary parasitoids from Argentina and Paraguay) were only found associated to *H. pungens* sensu* stricto* and the ArAuPr‐C clade (Triapitsyn et al., 2018). The influence of such differential ecological interactions with natural enemies might be affecting patterns of genetic divergence in this species complex.

Results of phylogenetic and species delimitation analyses were not entirely congruent. Analysis with mtDNA visualized four major clades, whereas genome‐wide SNPs allowed to detect five well supported clades (Figure 3). The main difference being that *Hypogeococcus* mealybugs from northeastern Brazil, Puerto Rico, and United States (BrPRUS‐AP clade) and *H. pungens* sensu stricto (Ar‐A) were different species according to nuclear SNPs, while these clades appeared collapsed in the same group with mtDNA data (Figure 3). Such mito‐nuclear inconsistencies have often been reported in insects (Hinojosa et al., 2019; Weigand et al., 2017). Even though the mtDNA has been useful to trace the evolutionary history in many species groups (Ball & Armstrong, 2006; Hebert, Penton, Burns, Janzen, & Hallwachs, 2004), incomplete lineage sorting and past hybridization events may obscure species delimitation based only on mtDNA data, particularly in recently diverged taxa (Després, 2019; Hickerson, Meyer, & Moritz, 2006; Hinojosa et al., 2019). For instance, Moreyra et al. (2019) reported a mitogenomic study in a cluster of closely related cactophagous *Drosophila* spp. inhabiting the southern cone of South America and found that the evolutionary history inferred by mitogenomes is not completely concordant with the phylogeny depicted by nuclear genomes (Hurtado, Almeida, Revale, & Hasson, 2019). The authors proposed that either incomplete lineage sorting (ILS) and/or introgressive hybridization could account for the pattern observed. A word of caution is needed before arriving at definitive conclusions in the present study due to limitations of the mtDNA dataset, that consisted of only a few hundred base pairs of the mtDNA gene encoding *COI*. In contrast, the evolutionary history depicted by the nuclear genomic dataset may be considered more reliable since it consisted of more than one thousand widely distributed SNPs.

Phylogenetic trees based on either nuclear SNPs or mtDNA indicated that feeding on Amaranthaceae was the more likely ancestral condition. However, results yielded by mtDNA and SNPs datasets were not completely congruent concerning the evolution of host plant use; both cactus feeding species formed a derived monophyletic clade in the tree based on nuclear SNPs, while cactophagy appeared to have evolved twice in the tree obtained with mtDNA data. However, to unveil the ancestral host in these clades and to evaluate the evolution of host plant use and the biogeographic history of the genus in the continent, other well‐delimited cactophagous species of the genus *Hypogeococcus* from South America, such as *Hypogeococcus spinosus* Ferris and *H. festerianus*, should be included in an expanded analysis.

### 4.2 Puerto Rican cactus mealybugs derive from southeastern Brazilian cactus feeding populations

Our analyses, based on nuclear SNPs and mtDNA, allowed us to establish that the Puerto Rican cactus pest derives from a population similar to the southeastern Brazilian cactus feeding clade (Figures 2 and 3). In our previous study using only mtDNA and restricted sampling in the native range (Argentina), the Puerto Rican cactus pest clustered close to *H. pungens* sensu stricto (Poveda‐Martínez et al., 2019). However, when we expanded the sampling to Paraguay and along the southeast and northeastern Atlantic coast of Brazil and extended the genetic sampling to genome‐wide SNPs, we found that the Puerto Rican cactus pest fell into the same cluster with populations from southeastern Brazil (Figure 2). Moreover, samples collected on Cactaceae in southern California shared the same and unique mtDNA haplotype with the Puerto Rican cactus pest. This finding is a warning of the threat that the presence of this pest represents to cactus diversity in the United States and Mexico, where cactus diversity is high. Since the first detection of the pest in Puerto Rico in 2005 (Segarra‐Carmona, Ramírez‐Lluch, Cabrera‐Asencio, & Jiménez‐López, 2010), the mealybug now attacks half of the 14 native Puerto Rican cactus species (including three endemic and two endangered species) occurring in dry forests, causing large gall‐like tissue deformations that often lead to high plant mortality (Carrera‐Martínez, Aponte‐Díaz, Ruiz‐Arocho, & Jenkins, 2015; Triapitsyn et al., 2020). With the new record of the pest in California, the pest has spread beyond its current distribution range to potential cactus hosts throughout North America (including Mexico) and the Caribbean. Identification of the source of the cactus pest that invaded Puerto Rico and the southern United States is an important accomplishment since it may help to develop more specific biological control strategies aimed at protecting wild cactus from this mealybug. In classical biological control programs, the correct identification of the target species is a key issue for searching for natural enemies of the pest in its native area (Hoelmer & Kirk, 2005). Thus, untangling the evolutionary history of the *H. pungens* species complex did not only have taxonomic and systematic relevance, but from a practical perspective, it indicated that the design of biological control strategies (e.g., search for natural enemies) against the pest should focus on southeastern Brazilian cactus feeding mealybugs.

Our results showed that Amaranthaceae‐ and/or Portulacaceae‐feeding mealybugs collected in Puerto Rico and continental United States were also derived from Amaranthaceae‐ and/or Portulacaceae‐feeding *Hypogeococcus*, though, in this case, from northeastern Brazil (Figures 2 and 3). These findings suggested two invasion events for mealybugs of the *H. pungens* species complex into Puerto Rico and the continental United States, one involving cactus feeders and the other mealybugs feeding on Amaranthaceae and/or Portulacaceae. Comparisons of the levels of genetic diversity in the native and invasive ranges of these mealybugs supported the idea of the occurrence of founder events during the colonization of Puerto Rico and southern United States. In both cases, introduced populations showed lower levels of genetic diversity in both mtDNA and nuDNA than in the respective native ranges, suggesting that a reduced number of individuals were involved in each colonization process (Table 3). Many invasive species have been capable of thriving in novel environments despite the reduction of genetic variation as a consequence of founder events that may negatively impact fitness, survival, and evolutionary potential of the invasive populations (Koch et al., 2020; Logan, Minnaar, Keegan, & Clusella‐Trullas, 2020; Tsutsui, Suarez, Holway, & Case, 2000). In this vein, two putative species of the *H. pungens* complex have been successful invaders and continue to spread throughout the Caribbean and southern United States, threatening native species and cactus diversity despite the loss of genetic variation.

## CONFLICT OF INTEREST

The authors declare that they have no conflict of interest.

## AUTHOR CONTRIBUTION

**Daniel Poveda‐Martínez:** Conceptualization (equal); Data curation (lead); Formal analysis (lead); Investigation (lead); Methodology (lead); Project administration (equal); Supervision (equal); Validation (equal); Visualization (equal); Writing‐original draft (lead); Writing‐review & editing (equal). **María Belén Aguirre:** Conceptualization (equal); Data curation (equal); Formal analysis (equal); Investigation (equal); Methodology (equal); Supervision (equal); Validation (equal); Writing‐original draft (equal); Writing‐review & editing (equal). **Guillermo Logarzo:** Conceptualization (equal); Data curation (equal); Funding acquisition (lead); Investigation (equal); Project administration (lead); Resources (equal); Validation (equal); Writing‐original draft (equal); Writing‐review & editing (equal). **Stephen D. Hight:** Conceptualization (equal); Funding acquisition (lead); Project administration (equal); Supervision (equal); Validation (equal); Writing‐original draft (equal); Writing‐review & editing (equal). **Serguei Triapitsyn:** Project administration (equal); Resources (equal); Supervision (equal); Validation (equal); Visualization (equal); Writing‐review & editing (equal). **Hilda Diaz‐Soltero:** Funding acquisition (equal); Investigation (equal); Project administration (equal); Supervision (equal); Validation (equal); Writing‐review & editing (equal). **Marcelo Diniz Vitorino:** Data curation (equal); Methodology (equal); Supervision (equal); Validation (equal); Visualization (equal); Writing‐review & editing (equal). **Esteban Hasson:** Conceptualization (equal); Data curation (equal); Formal analysis (equal); Investigation (equal); Methodology (equal); Project administration (equal); Supervision (equal); Validation (equal); Writing‐original draft (equal); Writing‐review & editing (lead).

## AUTHOR CONTRIBUTION

D.P.M., M.B.A., G.L., S.D.H., and E.H.: Design. D.P.M. and M.B.A.: Research performance; analysis. M.B.A., E.H., G.L., S.D.H., M.V.D., S.V.T., and H.D.S.: Field collection. G.L., S.D.H., and H.D.S.: Funding. D.P.M., M.B.A., and E.H.: Writing first draft. all co‐authors contributed equally to the revisions.

## Supporting information

Supplementary Material

## Data Availability

Raw sequence data are available at the National Centre for Biotechnology Information (NCBI), under the BioProject accession number PRJNA593002. Reference genome is available as FuEDEI_HPun_1.1.fa in NCBI under accession number: JAAOIU000000000. New mtDNA haplotypes were deposited in GenBank under access numbers: MT138921 – MT138931.
