# Supplementary material for: Species complex diversification by host plant use in an herbivorous insect: The source of Puerto Rican cactus mealybug pest and implications for biological control
Source: Ecol Evol. 2020 Aug 20;10(19):10463–80. doi: 10.1002/ece3.6702 (PMC7548167; doi:10.1002/ece3.6702)

**Supporting information**

Species complex diversification by host plant use in an herbivorous insect: The source of Puerto Rican cactus mealybug pest and implications for biological control. D. Poveda Martínez, M.B. Aguirre, G.A. Logarzo, S.D. Hight, S. Triapitsyn, M. Vitorino, H. Diaz-Sotero, and E. Hasson

**Table of contents:**

**Table S1.** Pairwise Fst estimates for *Hypogeococcus pungens* species complex populations sampled in native and invaded areas.

**Table S2.** Pairwise Fst estimates for the groups defined by cluster analysis.

**Table S3.** Species delimitation scenarios for *Hypogeococcus pungens* species complex.

**Figure S1**. Outcomes of single locus species delimitation analyses.

**Supplemental Table 1.** Pairwise Fst (Weir & Cockerham 1984) estimates for *H. pungens* species complex populations sampled in native and non-native ranges based on 1707 SNPs (A) and mtDNA (B).

| **A. nextRAD** | | **Native** | | | | **Non-native** | |
| --- | --- | --- | --- | --- | --- | --- | --- |
|  |  | **ARA** | **ARC** | **BRC** | **BRA** | **PRC** | **PRA** |
| **Native** | **ARC** | 0.8274 |  |  |  |  |  |
|  | **BRC** | 0.8146 | 0.8278 |  |  |  |  |
|  | **BRA** | 0.7420 | 0.6152 | 0.6027 |  |  |  |
| **Non-native** | **PRC** | 0.8494 | 0.8841 | 0.2827 | 0.6623 |  |  |
|  | **PRA** | 0.6892 | 0.9626 | 0.9523 | 0.3083 | 0.9698 |  |
|  | **USA** | 0.6736 | 0.9547 | 0.9407 | 0.2310 | 0.9599 | 0.0785 |
|  | | | | | | | |
| **B. mtDNA** | | **ARA** | **ARC** | **BRC** | **BRA** | **PRC** | **PRA** |
| **Native** | **ARC** | 0.7360 |  |  |  |  |  |
|  | **BRC** | 0.6996 | 0.7989 |  |  |  |  |
|  | **BRA** | 0.4379 | 0.6321 | 0.6068 |  |  |  |
| **Non-native** | **PRC** | 0.8288 | 0.8449 | 0.4118 | 0.6909 |  |  |
|  | **PRA** | 0.2385 | 0.7836 | 0.8292 | 0.5053 | 0.9524 |  |
|  | **USA** | 0.5326 | 0.8005 | 0.8977 | 0.5742 | 0.9999 | 0.5000 |

*ARA, Argentina mealybugs feeding on Amaranthaceae; ARC, Argentina, Australia and Paraguay mealybugs feeding on Cactaceae; BRA, Brazil mealybugs on Amaranthaceae; BRC, Brazil mealybugs on Cactaceae; PRC, Puerto Rico mealybugs on Cactaceae: PRA, Puerto Rico mealybugs on Amaranthaceae; USA, The US mealybugs on Amaranthaceae.

**Supplemental Table 4.** Pairwise Fst (Weir & Cockerham 1984) estimates for the groups defined by cluster analysis in *H. pungens* species complex from native and non-native range based on 1707 SNPs data.

| **Fst-Clusters** | **ArPaAu-C** | **BrPR-C** | **BrPRUS-AP** | **Br-A** |
| --- | --- | --- | --- | --- |
| **Ar-A** | 0.7592 | 0.7696 | 0.5670 | 0.7649 |
| **ArPaAu-C** |  | 0.7586 | 0.9155 | 0.9097 |
| **BrPR-C** |  |  | 0.9062 | 0.9026 |
| **BrPRUS-AP** |  |  |  | 0.9452 |

Ar-A clade: formed by Argentina mealybugs feeding on Amaranthaceae; ArPaAu-C clade: specimens from Argentina, Paraguay and Australia feeding on Cactaceae; BrPR-C clade: specimens from southern Brazil and Puerto Rico feeding on Cactaceae; BrPRUS-AP clade: specimens from northern Brazil, Puerto Rico and the USA feeding on Amaranthaceae and/or Portulacaceae; Br-A clade: specimens from southern Brazil feeding on Amaranthaceae.

- Weir, B.S., & Cockerham, C.C. (1984). Estimating F-statistics for the analysis of population structure. *Evolution*, 38, 1358–1370.

**Supplemental Table 5.** Results of Species delimitation analyses for *H. pungens* species complex assuming two models. The table shows the different species delimitation models evaluated along with the Bayes Factor species Delimitation method (BDF*).

| **Models** | **description** | **n species** | **MLE** | **Bayes factor** | **Rank** |
| --- | --- | --- | --- | --- | --- |
| **4 species** | as major mitochondrial clade | 4 | -21919.28743 | 546.11 | 2 |
| **5 species** | **as major genome cluster** | **5** | **-21646.23** | **-546.11** | **1** |

*The table shows the different species delimitation models evaluated with Bayes Factor Species Delimitation method (BFD*) (Leaché, Fujita, Minin, & Bouckaert, 2014). Both models were analyzed based on the SNPs dataset. MLE, Marginal likelihood estimates. The best delimitation scenario is shown in bold.

- Leaché, A. D., Fujita, M. K., Minin, V. N., & Bouckaert, R. R. (2014). Species delimitation using genome-wide SNP data. Systematic Biology, 63(4), 534-542.

**Supplemental Figure 1.** Outcomes of single locus species delimitation methods, the Generalized Mixed Yule Coalescent (GYMC) and Bayesian Poisson Tree Processes (bPTP). Both analyses were based on the mtDNA dataset.


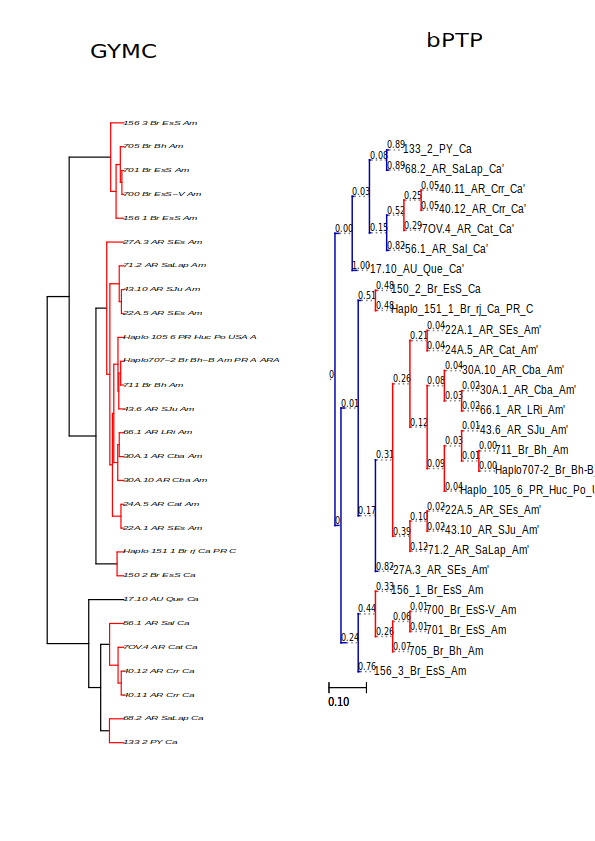

Supplement: Supplementary file 1 — Supplementary Material [file ECE3-10-10463-s001.docx]
